# The complete mitochondrial genome of *Fruhstorferiola tonkinensis* (Orthoptera: Catantopidae)

**DOI:** 10.1080/23802359.2016.1180555

**Published:** 2016-07-06

**Authors:** Xinmei Zhang, Liliang Lin

**Affiliations:** College of Life Sciences, Shaanxi Normal University, Xi'an, China

**Keywords:** *Fruhstorferiola tonkinensis*, mitochondrial genome, phylogeny

## Abstract

*Fruhstorferiola tonkinensis* belongs to the group of Catantopidae, which is the largest group in Acridoidae. The complete mitochondrial genome (mitogenome) of *Fruhstorferiola tonkinensis* is 15,637 bp in length, including 13 protein-coding genes (PCGs), 22 transfer RNA (tRNA) genes, 2 ribosomal RNA genes and 1 A + T-rich region. The gene order and arrangement are identical to other Acridoidae species. Most PCGs (except for the ATP6) start with the typical ATN codons, while most stop codons are TAA, TAG. The combined dataset of 13 PCGs and 2 rRNAs from 17 grasshoppers (including 15 Catantopidae species and 2 outgroups) is used to construct phylogenetic tree and analyse phylogenetic relationship of Catantopidae.

Animal mitogenome is a circular, double-strand molecule of 15–20 kb in size (Ye et al. 2012). It generally includes 37 genes, 13 PCGs, 22 tRNAs, 2 rRNAs. It also contains a non-coding region (A + T-rich region) in the insect. The specimens of the *Fruhstorferiola tonkinensis* were collected by clap net in Xingan, Guangxi province, China in June 2005. They were stored at 100% ethanol, and -4 °C in the College of Life Science in Shaanxi Normal University. The mitogenome of *Fruhstorferiola tonkinensis* is 15,637bp in length, and has been deposited in GenBank (accession no. KU942377), containing 13 PCGs, 22 tRNAs, 2 rRNAs and one A + T rich region. The overall base composition of the complete mitogenome was A (42.55%), T (32.75%), C (14.29%), G (10.14%). All 13 PCGs of the mitogenome are recognized by Spin program in Staden Package combined with other Orthopterans species (Staden et al. 1998). The initiation codons and termination codons of the 13 PCGs in *Fruhstorferiola tonkinensis* were identical to other orthopterans species (ATN and TAA/TAG). The 22 tRNAs were predicted by online software tRNA-Scan-SE (Lowe & Eddy 1997), and has identical clover-leafs structure (Stewart & Beckenbach 2006) except for the tRNA^Ser (AGN)^. The two rRNA genes (16S rRNA and 12S rRNA) were located between tRNA^Leu(CUN)^ gene and the control region, and separated by tRNA^Val^ gene. The length of 16S rRNA and 12S rRNA were respectively determined to be 1315 bp and 797 bp. The A + T-rich region was located between 12S rRNA and tRNA^Ile^ gene, and was 805 bp in size.

Phylogeny of Catantopidae was mostly studied based on single genes or several gene fragments (Ma & Huang 2006; Pan et al. 2006). However, a phylogeny inferred from the complete mitogenome is more reliable (Yang et al. 2015). Nowadays, with the development and advancement of the sequence analysis, the complete mitogenome of many animals have been determined and used for phylogenemic analysis (Camerson 2014; Zhou et al. 2014). Based on the combined dataset of 13 PCGs and 2 rRNAs of the *Fruhstorferiola tonkinensis* and the other 16 taxa (including 14 taxa and 2 outgroups taxa), phylogenetic tree was constructed by PAUP version 4.0 beta10 (Swofford 2003) (Figure 1). From the tree topology, monophyly of subfamilies Cyrtacanthacridinae, Melanoplinae, Oxyinae were supported, while monophyly of Catantopinae was not supported. *Spathosternum prasiniferumlongipennis* was clustered at the base of Catantopidae, sistered to other Catantopidae species. Most phylogeny are identical to traditional taxonomy, but we need more taxon sampling to clearly understand phylogenetic relationships of Catantopidae.

**Figure 1.**
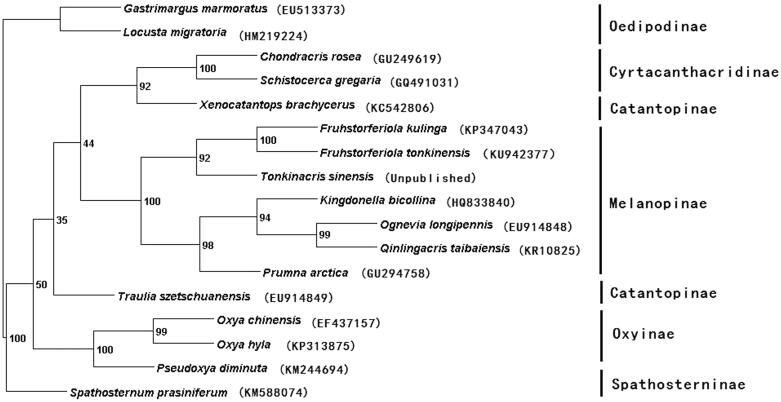
Phylogenetic relationship of the Catantopidae using mitochondrial PCGs and rRNAs combined dataset. (GenBank accession numbers are listed in parentheses after the species names).
